# Editorial: Gender-specific inequalities in the education system and the labor market

**DOI:** 10.3389/fsoc.2023.1254664

**Published:** 2023-07-24

**Authors:** Pia N. Blossfeld, Magdalena Pratter, Wilfred Uunk

**Affiliations:** ^1^Institute for Sociology, University of Innsbruck, Innsbruck, Austria; ^2^LIfBi, Bamberg, Germany

**Keywords:** inequality, gender, education, labor market, gender segregation

## Introduction

This Research Topic in Frontiers on gender-specific inequalities in education and the labor market aims to bring together recent empirical studies on differences in women's and men's educational and labor market preferences, choices, and opportunities. Existing studies have shown that women have caught up with and even surpassed men in educational attainment (Shavit and Blossfeld, 1993; Breen et al., 2010; Hadjar and Berger, 2011; DiPrete and Buchmann, 2013) and that women are increasingly participating in the labor market and in jobs with higher socio-economic status. Yet many questions about gender inequalities in education and the labor market remain unanswered, at least in the country contexts examined below. What are the consequences of educational expansion in the parental generation, which has been particularly strong for women, for the educational attainment of daughters and sons? Are daughters and sons more downwardly mobile in education today? Are men more likely to share household tasks with women when the educational gap between husbands and wives is smaller? Are occupations increasingly sex-segregated due to women's empowerment and self-expression? What role do gender ideology, educational aspirations, work values, and household assets play in this process, and can intensive counseling programs lead to more gender-atypical university major choices? Do boys and girls with an immigrant background exhibit higher transition probabilities to a more prestigious educational path than children without an immigrant background? And to what extent do men and women differ in the preferences of work arrangements?

These and other questions are addressed in this Research Topic. We focus first on issues related to gender and education and then on gender and the labor market.

## Gender and educational expansion

The first two articles in this Research Topic are devoted to the long-term effects of educational expansion on gender-specific differences in educational outcomes. It begins with a contribution by Blossfeld, who uses data from the National Educational Panel Study (NEPS) to analyze how intercohort improvements in family educational attainment contribute to the rising educational attainment of sons and daughters in West Germany. Her article aims to answer two questions: (1) Is the improvement in the educational attainment of families particularly beneficial to the rise in daughters' educational attainment, since families with higher educational attainment are generally considered more gender equitable, or have both sons and daughters benefited similarly, and (2) have daughters particularly benefited from mothers' catching up with fathers' educational attainment, since mothers in particular serve as role models for their daughters? Her empirical results suggest that both sons and daughters have benefited similarly from the intercohort change in family educational attainment. She also finds that maternal education is equally relevant for the educational opportunities of sons and daughters. In addition, she shows that educational downward mobility has increased for sons and daughters as the proportion of children from academic family backgrounds rises. The second contribution, by Nennstiel and Becker, also observes that absolute educational downward mobility has increased, using Swiss data from the Census, the Cumulative Structural Survey, and the Population and Household Statistics (STATPOP) from the Swiss Federal Statistical Office. As the share of privileged children increases, so does the pool of children who may be downwardly mobile. Their analysis, which compares birth cohorts 1951–1990 for Switzerland, also addresses relative intergenerational educational mobility. Nennstiel and Becker find that relative mobility rates have declined slightly for women and men. While there were gender differences in relative mobilities for the oldest cohorts studied, there is a convergence between the sexes across cohorts. For birth cohorts born after 1970, maternal education becomes more important for women's relative mobility than for men's, but remains less important than paternal education.

The third contribution by Peng and Wu addresses another consequence of the educational expansion process, namely how the reduction of the education gap between husbands and wives influences (in)equality in the division of housework in the Chinese context. The authors use the China Family Panel Studies (CFPS2018) and find that in households with a lower education gap between husband and wife, gender inequality in housework sharing is also lower. This effect of the education gap between husband and wife on inequality in household labor sharing is explained by the relative income and relative working hours of husband and wife.

## Gender and stem education

Although women have caught up with men in educational attainment, women still differ greatly from men in their subject choices in the educational system (Ware and Lee, 1988; Turner and Bowen, 1999; Bradley, 2000; Barone, 2011; Mann and DiPrete, 2013; Van de Werfhorst, 2017; Uunk et al., 2019; Jacob et al., 2020; Hägglund and Leuze, 2021). Women are much more likely to choose feminine subjects such as teacher education, humanities, social sciences, and healthcare. Men, on the other hand, are much more likely to opt for Science, Technology, Engineering or Mathematics (STEM). This horizontal gender segregation creates further gender inequalities, as STEM education offers better wage and career prospects than non-STEM education (Christie and Michael, 2001; Black et al., 2008; OECD, 2017).

In this Research Topic, three articles explore some of the possible causes of gender differences in the choices of STEM subjects in education. The first two articles focus on what is known as the Gender-Equality-Paradox (GEP). GEP is the puzzling finding that men and women in more affluent and gender-equal countries choose more gender-specific fields of studies than those in less developed countries (Bradley, 2000; Stoet and Geary, 2018, 2020; Richardson et al., 2020). In their paper, Erdmann, Hill et al. test GEP longitudinally by describing how adolescents' gender-specific occupational expectations change over time (2006–2018) and how female empowerment and cultural norms might influence gender-specific occupational expectations. Using data from two waves of the Programme for International Student Assessment (PISA), 2006 and 2018, from 26 European countries, the authors show that only in some countries occupational expectations became more segregated. In other countries, the proportion of gender parity or gender-atypical expectations increased. Moreover, in contrast to the cross-sectionally observed GEP, female empowerment and self-expression values led to less gender-typical occupational expectations among girls and boys. In his paper, Uunk also finds evidence against (interpretations of) GEP, using data from the PISA 2012 wave. Although wealthier countries show a larger male-favorable gap in STEM aspirations, multilevel analyses show that at the micro-level, household wealth is not associated with a larger gender gap in math intentions. Girls are also not less likely to choose math and STEM as household wealth increases.

The study by Gambaro et al. investigates the gender-typical occupational aspirations of immigrant and non-immigrant youth aged around 16 in four European countries (England, Germany, the Netherlands, and Sweden) using data from the Children of Immigrants Longitudinal Survey (CILS4EU). The authors find that immigrant boys and girls aspire to slightly less gender-typical occupations than their peers in the majority population. More ambitious educational aspirations, but not gender ideology and work values, partly explain these smaller gender differences in the occupational aspirations of children with immigrant backgrounds.

In the last paper on gender and STEM, interventions to reduce gender bias in subject choices are addressed. The contribution by Erdmann, Schneider et al. examines if and how intensive counseling programs can lead to a more gender-atypical major choice in higher education among men and women in North Rhine-Westphalia (Germany). Their study combined a panel study with an experimental design. They followed students 2 years before to 3 years after they completed a higher education entry certificate and randomly assigned students to a control or treatment group. Their results show that intensive counseling increased the gender-atypical degree choices of men and women. In particular, men were more likely to choose gender-atypical majors when they participated in the advising program. In addition, there is some evidence that the counseling program increases the likelihood that students will remain in their gender-atypical major (although this could not be measured directly).

## Gender, migration, and education

A final paper on education and gender in this Research Topic, focuses on immigration background and educational attainment. A well-established finding in education research is that children with an immigrant background have a higher probability of transitioning to a more prestigious educational track than children without an immigrant background, after controlling for previous educational achievement and socioeconomic status (Kristen and Dollmann, 2010; Jonsson and Rudolphi, 2011; Relikowski et al., 2012; Griga and Hadjar, 2014; Salikutluk, 2016; Dollmann and Weißmann, 2020). This ethnic choice effect is often explained by higher upward mobility aspirations among children from immigrant backgrounds (Kao and Tienda, 1995), a finding also central in the paper of Gambaro et al. in this Research Topic. Glauser and Becker aim to contribute to this literature by (1) examining whether there are gender differences in this ethnic choice effect and (2) investigating whether educational aspirations can explain these higher educational transition probabilities into more demanding educational tracks for male and female immigrant students alike. Glauser and Becker concentrate on migrant groups from the Balkans, Turkey, and Portugal, and their country of analysis is Switzerland. Using panel data from the Transitions from Education to Employment (TREE) and the Determinants of educational choices and vocational training opportunities (DAB) studies, they demonstrate that only male migrants exhibit higher transition probabilities to more demanding educational tracks when controlling for prior school performance and family background, and that aspirations mediate part of this ethnic choice effect.

## Gender and the labor market

Finally, empirical studies observe not only gender inequalities in education, but also in the labor market. Although women have increased their labor market participation dramatically in recent decades, women still earn less than men and work fewer hours (Rosenfeld and Kalleberg, 1990; Charles, 2011; England et al., 2020). In addition, women are still more likely to work in different occupations and perform different tasks than men (Steinmetz, 2011; Levanon and Grusky, 2016; Weeden, 2019; Zhu and Grusky, 2022). Two articles in this Research Topic address gender inequalities in the labor market. The paper by Jost and Möser investigates whether there are gender-specific preferences for work arrangements (part-time vs. full-time work, reductions of work, career advancement, salary, further training opportunities, collegial vs. competitive work environment, and flexibility) in Switzerland. In particular, they are interested in whether these gender-specific preferences are the result of gender-specific role expectations. They aim to test social role and human capital theory, both of which assume that gender roles are based on division of labor within couples. They conducted a discrete choice experiment that was included in the 10th wave of the DAB panel study. A first finding from their experiment is that women have a stronger preference for part-time work and a collegial work atmosphere than men and that men place more value on career prospects. In terms of gender role expectations between and within groups of men and women, they show that only men and women with more traditional gender role expectations (in terms of sharing housework and having children at a young age) are more likely to choose gender-typical job characteristics.

The final contribution by Folberg et al. examines gender differences in entrepreneurial interests using data from the University of Nebraska Medical Center. They test goal congruity theory, which assumes that people adopt gender-stereotypic goal orientations in response to social pressures to conform to traditional gender roles. In particular, they are interested in whether successful entrepreneurship is perceived as dominant but not described as typically female. In addition, they examine the role of (gender-stereotyped) agency, including the dimensions of competence, self-direction, dominance orientations, and community goal orientations (e.g., warmth) in female and male entrepreneurial interests, supplemented by sub-dimensions of gender stereotypes and stereotype-related constructs. Results indicate that entrepreneurship, although perceived as dominant, is not perceived as inherently masculine and can fulfill communal goals (e.g., caring for others). Both women and men tend to prefer careers that align with socially perceived gender roles, but they do not show differential interest in entrepreneurship.

## Author contributions

All authors listed have made a substantial, direct, and intellectual contribution to the work and approved it for publication.
